# Towards more efficient and robust evaluation of sepsis treatment with deep reinforcement learning

**DOI:** 10.1186/s12911-023-02126-2

**Published:** 2023-03-01

**Authors:** Chao Yu, Qikai Huang

**Affiliations:** 1 School of Computer Science and Engineering, Sun Yat-sen University, Guangzhou, China; 2 Fudan University Pudong Medical Center, Shanghai Pudong Hospital, Shanghai, China

**Keywords:** Deep reinforcement learning, Inverse learning, Sepsis, Intensive care units

## Abstract

**Background:**

In recent years, several studies have applied advanced AI methods, i.e., deep reinforcement learning, in discovering more efficient treatment policies for sepsis. However, due to a paucity of understanding of sepsis itself, the existing approaches still face a severe evaluation challenge, that is, how to properly evaluate the goodness of treatments during the learning process and the effectiveness of the final learned treatment policies.

**Methods:**

We propose a deep inverse reinforcement learning with mini-tree model that integrates different aspects of factors into the reward formulation, including the critical factors in causing mortality and the key indicators in the existing sepsis treatment guidelines, in order to provide a more comprehensive evaluation of treatments during learning. A new off-policy evaluation method is then proposed to enable more robust evaluation of the learned policies by considering the weighted averaged value functions estimated until the current step.

**Results:**

Results in the MIMIC-III dataset show that the proposed methods can achieve more efficient treatment policies with higher reliability compared to those used by the clinicians.

**Conclusions:**

A more sound and comprehensive evaluation of treatments of sepsis should consider the most critical factors in infulencing the mortality during treatment as well as those key indicators in the existing sepsis diagnosis guidelines.

## Background

Defined as severe infection causing life-threatening acute organ failure, sepsis is a leading cause of mortality and associated healthcare cost in critical care [1]. According to the latest report from the World Health Organization, in 2017 there were 48.9 million cases of sepsis and 11 million sepsis-related deaths worldwide, accounting for almost $20\%$ of all global deaths [2]. While a large number of international organizations have devoted significant efforts to provide general guidance over the past 20 years, physicians at practice still lack universally agreed-upon decision support for sepsis treatment. This dilemma has intrigued an increasing interest in applying advanced machine learning and data analysis methods to deduce more efficient treatment policies for sepsis patients. Particularly, *Reinforcement Learning* (RL) [3] has emerged as a promising solution due to its capability of addressing treatment problems characterized with a sequential decision making process and evaluative delayed feedbacks [4, 5].

There are a number of studies that have applied RL in deriving more efficient treatment policies for sepsis in the past years, utilizing algorithms such as model-based *Policy Iterations* (PI) [6, 7], *Deep Deterministic Policy Gradient* (DDPG) [8], *Dueling Double Deep Q-Networks* (DDDQN) [9, 10] and *Proximal Policy Optimization* (PPO) [11]. Usually, RL could discover treatment policies that resemble those by the clinicians most of the time, yet sometimes suggest novel policies that are more efficient but rarely adopted by clinicians in practice. While comprehensive qualitative and quantitative evaluations have been conducted to verify the benefits of RL-driven policies, there is still an on-going debate on whether the evaluation is sound enough to support the claims of effectiveness and robustness of the derived treatment policies [12].

Evaluation in this type of medical decision making normally has two aspects of interpretations. First, to enable the functionality of RL algorithms, providing an accurate evaluation of the actions (i.e., treatments) during learning is of great importance. This issue stems from the reward formulation problem in general RL research, which is exaggerated in healthcare domains as there are normally numerous indexes that can potentially influence the therapeutic decisions, and it is usually unclear which indexes are the most critical and what different roles of these indexes can play in consisting of a reward function that lead to the final treatment performance. The other crucial issue is the evaluation of the final learned treatment policies. Due to high cost of experiments and uncontrolled risks of treatments, it is infeasible to estimate the policy performance by running it directly on the patients. Thus, it is needed to estimate how the learned policies might perform on retrospective data before testing them in real clinical environments. The task of estimating the performance of some evaluation policy given data generated by a different behavior policy is known as the challenging *off-policy evaluation* (OPE) problem that has been widely investigated in the RL community [13]. In medical settings, the OPE problem becomes even more tricky, since many factors such as state representations, estimator variance and confounders would result in unreliable or even misleading evaluation of the quality of a treatment policy [14, 15]. As such, how to develop more robust OPE methods is the key issue to guarantee the success of RL methods in healthcare applications.

In this work, we address the above evaluation problems in sepsis treatment by first proposing a deep inverse RL with Mini-tree *(DIRL-MT)* model to infer the potentially best reward functions from retrospective real medical data. In the model, the MT component discovers the most critical factors in influencing the mortality during treatment, while the DIRL component infers the complete reward function consisting of those critical factors and key indicators in the existing sepsis diagnosis guidelines. In this way, a more sound and comprehensive evaluation of treatments during learning can be made through mining the inherent treatment-mortality patterns from retrospective data and utilizing the prior domain knowledge from clinical practice. We empirically evaluate the proposed DIRL-MT model in the administration of *intravenous* (IV) and maximum *vasopressor* (VP) for sepsis patients using in the MIMIC III dataset [16]. Results show that the learned policy can reduce mortality compared to those given by the clinicians by a large margin. As our second contribution, we propose a new estimator, the *dueling weight* (DW), to reduce the variance of general OPE estimators. Unlike the existing estimators that only consider the value estimation at current time step, DW uses the difference of estimation between the past average value function and the current value function to represent the model estimation, and thus can incorporate learning information in a longer horizon into the model estimation process in order to obtain a more accurate model for variance reduction. We theoretically prove the upper bound bias and lower variance of DW, and experimentally verify its effectiveness in the sepsis treatment problem.

## Related works

RL has also been applied to solve the sepsis treatment problem by a number of studies in recent years. Komorowski et al. [6] applied model-based policy iteration in a discrete state and action space to learn the sepsis treatment strategy. Raghu et al. [9, 10] directly trained the policy in continuous state space using the *Dueling Double DQN* method. The authors [11] estimated the transition model in a continuous state space, and applied direct policy optimization methods to derive a treatment strategy. Li et al. [17] provided an online partially observable MDP method to take into account uncertainty and history information in sepsis treatment. Utomo et al. [18] used Monte Carlo to generate a real-time treatment recommendation and proposed a graphical model to show transitions of patient health conditions and treatments for better explainability. Peng et al. [19] applied the mixture-of-experts framework [20] in sepsis treatment by automatically switching between kernel learning and DRL depending on patients’ treatment history. More recently, Liu et al. [21] combined model-based and model-free RL policies for more efficient sepsis treatment by dynamically switching between these two policies depending on the states of patients. However, all these studies relied on some numerical reward functions that must be explicitly defined a priori to indicate the goal of treatments. On the contrary, our work applied IRL methods to infer the reward functions of clinicians during their treatment process. The benefits of IRL methods lie in the dynamic estimate of different factors that should be considered to evaluate the decision making performance. Moreover, unlike the existing works that directly used the normal OPE methods to evaluate the performance of the final learned policies, we proposed a new OPE estimator to reduce the variance of general OPE estimators.

## Methods

### Notations

The sepsis treatment problem can be modeled as a sequential decision making problems by episodic MDPs with a finite horizon, which can be defined by a tuple $\langle S, A, P, R, \gamma \rangle$, where *S* and *A* are the state and action space, $P : S\times A \times S \rightarrow {\mathbb {R}}$ is the transition function with $P(s_{t+1}|s_t, a_t)$ defined as the probability of reaching state $s_{t+1}$ after taking action $a_t$ in state $s_t$ at time *t*, $R : S\times A \rightarrow {\mathbb {R}}$ is the mean reward function with $R(s_t, a_t)$ defined as the immediate received reward after taking action $a_t$ in state $s_t$, and $\gamma \in [0, 1]$ is the discount factor. A (stationary) policy $\pi : S \times A \rightarrow [0, 1]$ is a stochastic mapping from states to actions, with $\pi (a_t|s_t)$ being the probability of taking action $a_t$ in state $s_t$. Let $\mu$ be the initial state distribution. The distribution of a *T*-step trajectory $\xi =(s_0, a_0, r_0\ldots ,s_{T-1}, a_{T-1}, r_{T-1}, s_T)$ is denoted as $P_{\xi }^{\pi }$, or simply as $\xi \sim (\mu , \pi )$. We use interchangeably $E_{\xi \sim (\mu , \pi )}$, $E_{P_{\xi }^{\pi }}$, or $E_{\xi }^{\pi }$ to denote the expectation over trajectory distributions. Meanwhile, the *T*-step discounted value of $\pi$ is defined as: $\upsilon ^{\pi }_{T} = E_{\xi \sim (\mu , \pi )}[\sum _{t=1}^{T} \gamma ^{t-1} r_t]$, where $s_0 \sim \mu$ and $r_t$ has mean value of $R(s_t, a_t)$ conditioned on $(s_t, a_t)$. When the value of $\pi$ is conditioned on $s_0 = s$ (or $a_0 = a$), the future expected value of a state (and an action) is expressed as $V^{\pi }_{T}(s)$ (and $Q^{\pi }_{T}(s,a)$). If *T* is of order $O(1/(1-\gamma ))$, then $\upsilon ^{\pi }_{T}$ approximates the infinite-horizon performance $\upsilon ^{\pi }_{\infty }$ [22]. When the true parameters of the MDPs are known, the value of the target policy can be computed by the Bellman equations: $V_t(s_t) = E_{a_t \sim \pi (.|s_t)}[Q(s_t, a_t)]$ and $Q_t(s_t,a_t) = E_{s_{t+1} \sim \pi (.|s_t, a_t)}[R(s_t, a_t)+\gamma V_{t}(s_{t+1})]$.

There are a set of *T*-step trajectories $M = {\xi (i)}_{i=1}^n$ generated by a fixed stochastic policy $\pi _b$, known as the behavior policy. The goal of OPE is to find an estimator ${\widehat{\upsilon }}^{\pi _e}_T$ that makes use of the data generated from running $\pi _b$ to estimate the performance of another evaluation policy $\pi _e$. The estimator will have good performance if it has low mean square error (MSE), i.e., $MSE = E_{P_{\xi }^{\pi _b}}[({\widehat{\upsilon }}^{\pi _e}_T-\upsilon ^{\pi _e}_T)^2]$, where ${\widehat{\upsilon }}^{\pi _e}_T$ and $\upsilon ^{\pi _e}_T$ denote an estimated value and the real value of $\pi _e$, respectively.

One major type of approaches is *Importance Sampling* (IS) that uses a *cumulative importance ratio* term to correct the mismatch between the distributions under the behavior policy and the target policy [23]. In the IS estimator, the performance of $\pi _e$ can be expressed as the mean of *n* trajectories: $V_{IS}^{\pi _e}=\frac{1}{n}\sum _{k=1}^{n}V_{IS}^{\pi _e(k)} =\frac{1}{n}\sum _{k=1}^{n} \sum _{t=1}^{T} \omega _{0:t}^{(k)} \gamma ^t r_t^{(k)}$, where $\omega _{0:t}^{(k)}= \prod _{t=0}^T \frac{\pi _e(a_t|s_t)}{\pi _b(a_t|s_t)}$ is *cumulative importance ratio* of the *k*th trajectory, and $r_t^{(k)}$ is the expected reward function at time *t* of the *k*th trajectory. Since IS corrects the difference between $\pi _b$ and $\pi _e$ based on the accumulated reward along the whole trajectory, it can provide unbiased estimate of the value of $\pi _e$. However, IS methods are notorious for its high variance, especially when there is a big difference between the distributions of the evaluation and behavior policies, and the horizon of the RL problem goes long [24]. *Doubly Robust* (DR) methods are then proposed by adding estimated value functions into the IS estimator in order to achieve low variance of IS and low bias of model-based methods [22]. In the DR estimator $V_{DR}^{\pi _e}=\frac{1}{n}\sum _{k=1}^{n}V_{DR}^{\pi _e(k)}$, where $V_{DR}^{\pi _e(k)} = {\widehat{V}}(s)+\frac{\pi _e(a|s)}{\pi _b(a|s)}(r-{\widehat{R}}(s, {a}))$. Here, ${\widehat{V}}(s)= E_{a \sim \pi _b}[\frac{\pi _e(a|s)}{\pi _b(a|s)} {\widehat{R}}(s, {a})]$, and ${\widehat{R}}(s, {a})$ is an estimate of the observed stochastic return *r*, and can be estimated possibly by performing regression over the *n* *T*-step trajectories. Provided ${\widehat{R}}(s, {a})$ is a good estimate of *r*, the magnitude of $r-{\widehat{R}}(s, {a})$ can be much smaller than *r*, which can lead to lower variance of the DR estimator compared to IS. Omitting the notation of trajectory *k* hereafter, the single-step updated formula of DR can be extended to sequential settings as $V_{DR}^{T-t+1} := {\widehat{V}}(s_t)+\omega _{0:t}(r_t+\gamma V_{DR}^{T-t}-{\widehat{Q}}(s_t, a_t))$ [22]. While several extensions to DR have been proposed in recent years [25], the DR estimators still face the problem in general model-based estimators regarding how well the value functions can be estimated.

To lower the variance of IS, a biased but consistent estimator *Weighted Importance Sampling* (WIS) [26] is proposed. For each trajectory, the estimates given by the step-wise WIS are $V_{step-WIS}^{\pi _e} =\sum _{t=0}^T \frac{\omega _{0:t}^{(k)}}{\omega ^{WIS}_t} \gamma ^t r_t^{(k)}$, where $\omega ^{WIS}_t =\sum _{k=1}^{n} \omega _{0:t}^{(k)}/n$ denotes the average cumulative important ratio at horizon *t*. Similarly, the DR can also be improved by defining $\omega ^{WDR}$ so as to obtain the step-wise *Weighted Doubly Robust* (WDR) estimator as $V_{WDR}^{T-t+1} = {\widehat{V}}(s_t)+\sum _{t=1}^T \omega ^{WDR}(r_t + \gamma V_{DR}^{T-t}-{\widehat{Q}}(s_t, a_t))$.

### Data acquisition and preprocessing

**Table 1:** Basic information statistics for patients that fulfilled the sepsis criteria

	%Male	Mean age	Total persons	Mortality ratio
Survivors	56.92	61.17	11980	14.5%
Non-survivors	55.70	67.95	2032	
Total-patients	56.77	62.01	14012	

Historical data of 14012 patients were obtained from the multi-parameter intelligent monitoring in intelligent care (MIMIC-III v1.4) database [16], excluding those admissions who were under the age of 18, or obtained the failed treatment process. The summary information about the patients is shown in Table 1.

We use seven different machine learning methods to fit the physiological measured values at different measurement times, including support vector machine (SVM), k-nearest neighbor (KNN), decision tree regressor (DTR), logistic regression (LR), gradient boosting tree (GBDT), extra trees regressor (ETR), and random forest regressor (RFR). The results of the corresponding loss values are shown in Table 2. We finally use ETR to fit historical data of every patient.

**Table 2:** Comparison of loss values of seven different machine learning methods in different physiological characteristics

Features	SBP	RR	GCS	MAP	HeartRate	DBP	MBP
$SVM(C=30.0)$	80.38	6.15	0.32	173.24	14.07	31.39	173.24
*KNN*	112.48	11.40	1.29	187.28	23.61	40.13	187.28
*DTR*	133.19	7.01	0.13	211.81	14.61	46.93	211.81
$LR(C=30.0)$	309.59	34.29	3.01	334.19	40.31	102.86	334.19
*GBDT*	107.67	7.50	0.16	187.07	17.40	38.77	187.07
*ETR*	89.09	5.36	0.17	154.38	13.95	30.99	157.57
*RFR*	92.52	6.57	0.40	146.31	14.00	31.39	139.06

After preprocessing, we can obtain complete data for each patient and take 1 hours as a timestep interpolation on a patient’s historic trajectory, from admission to discharge.

### The DIRL-MT model

**Table 3:** Definition of different reward functions

Indicator criterion	Rewards
*Sepsis*3.0	$Reward_{3.0}=\sum _{i=0}^1W_itanh(S_i)$
$Sepsis3.0^+$	$Reward_{3.0^+}=\sum _{i=2}^3W_itanh(S_i)$
*Sepsis*4.0	$Reward_{4.0}=\sum _{i=4}^5W_itanh(S_i)$
$Sepsis(3.0+3.0^+)$	$Reward_{3.0+3.0^+}=reward_{3.0}+reward_{3.0^+}$
$Sepsis(3.0+4.0)$	$Reward_{3.0+4.0}=reward_{3.0}+reward_{4.0}$
$Sepsis(3.0^++4.0)$	$Reward_{3.0^++4.0}=reward_{3.0^+}+reward_{4.0}$
*Sepsis*(*all*)	$Reward_{all}=reward_{3.0}+reward_{3.0^+}+reward_{4.0}$

We focus on RL solutions to derive more efficient policies for *intravenous* (IV) fluids and *maximum Vasopressor* (VP) management through inferring the possibly optimal reward functions during learning. To this end, we define a $5\times 5$ action space for the medical treatments covering the space of IV fluids and maximum VP in a given one hour window. This action space ranges from zero to the maximum allowed IV fluids and VP. A patient’s state is composed of 30 features from the items of Demographics, Lab Values and Vital Signs in the MIMIC-III database. To define a clinically guided reward function, a possible way is to use the existing criteria for diagnosing sepsis to indicate how the patient’s conditions have improved after a certain treatment has been conducted. Positive rewards should be given at intermediate timesteps for improvements in a patient’s wellbeing, and negative rewards for deterioration. Previous studies, e.g. [9, 11], defined the rewards on severity scores, such as SOFA and lactate levels, by penalizing high SOFA scores and lactate as well as increases in SOFA score and lactate. Considering the indicators for diagnosing septic shock in the third international consensus definitions for sepsis and septic shock (Sepsis-3) [27], we similarly define several different reward functions in Table 3, where the parameters $W_i$ are the weights of different indicators. In specific, $reward_{3.0}=\sum _{i=0}^1W_itanh(S_i)$, where $S_0=S_{t}^{QSOFA}-S_{t+1}^{QSOFA}$ denotes the variation of Quick *Sequential Organ Failure Assessment* (SOFA), and $S_1=S_{t}^{SOFA}-S_{t+1}^{SOFA}$ is the variation of SOFA, while ${reward}_{3.0^+}=\sum _{i=2}^3W_itanh(S_i)$ indicates the indicators for diagnosing septic shock, where $S_2=S_{t+1}^{MAP}-S_t^{MAP}$ and $S_3=S_{t+1}^{Lactate}-S_{t}^{Lactate}$. However, all these indicators only reflect the best known clinical practice that might be far from being optimal, and represent short-term treatment effect that is not necessarily correlated with the final mortality outcome.

**Fig. 1:**
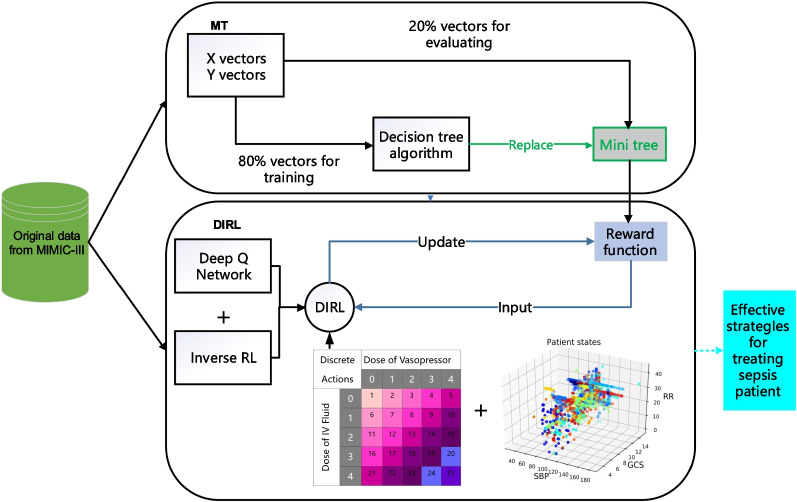
Overview of the DIRL-MT model

In order to provide a more comprehensive evaluation of the treatments during learning, we propose the DIRL-MT model in Fig. 1, where the MT component discovers the most critical indicators in affecting the long-term outcome of mortality, and the DIRL component infers the correlation among different indicators and learns the treatment policies by dynamically adapting the weights of the indicators in the reward function. The MT component in the experiments discovers that the *Partial Pressure of Oxygen* (PaO2) and *Prothrombin Time* (PT) are the most important indicators in influencing sepsis mortality. As such, we define a new reward function $reward_{4.0}$ as weighted sum of $S_4=S_{t+1}^{PaO2}-S_t^{PaO2}$ and $S_5=S_{t+1}^{PT}-S_t^{PT}$ that represent the variation of PaO2 and PT, respectively. Then, several combination of reward functions can be defined by combing the corresponding indicators as shown in Table 3. Particularly, combining the critical indicators from MT with some key indicators in the existing sepsis diagnosis guidelines (e.g., $reward_{3.0+4.0}$) can thus strike a balance of treatment evaluation between short-term effect and long-term mortality.
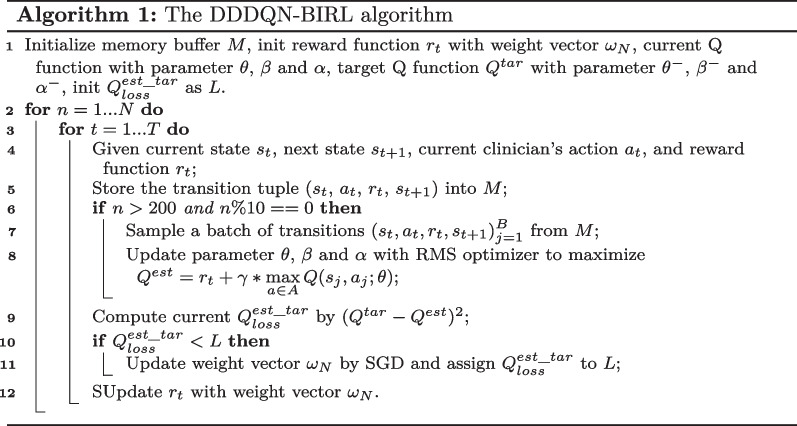


Algorithm 1 gives a detailed process of the DIRL component, using the *Dueling Double Deep Q Network* (DDDQN) [28, 29] for policy learning and the *Bayesian Inverse RL* (BIRL) to infer the optimal reward function (*i.e.*, updating the weights of reward indicators). More specifically, DIRL continuously minimizes the loss ($Q_{loss}^{est\_tar}$) between estimated Q_value ($Q^{est}$) and the target oriented Q_value ($Q^{tar}$) over time horizon *T* by Eq. (1),

1 $$\begin{aligned} Q_{loss}^{est\_tar}=\mathop {argmin}\limits _{\theta ; \beta , \alpha } \frac{1}{T} \sum _{t=1}^T {\left( Q^{est}-Q^{tar}\right) ^2}. \end{aligned}$$

where

2 $$\begin{aligned} \displaystyle \begin{aligned} Q_{t+1}^{tar}=r_{t+1}\!+\! \gamma Q\left( s_{t+1}, \mathop {argmax}\limits _{a \in A}\left( Q_{t+1}^{est}\right) ,\left( \theta _t^-; \beta _t^-,\alpha _t^-\right) \right) , \end{aligned} \end{aligned}$$

and

3 $$\begin{aligned} Q_{t+1}^{est}=V\left( s_{t+1};\theta _t,\beta _t\right) +A\left( s_{t+1},a;\theta _t,\alpha _t\right) \end{aligned}$$

represent the *Q* values updated using Double DQN and the Dueling DQN network, respectively.

### The DW estimator

We propose a new OPE estimator, the *Dueling Weight* (DW), in order to provide a more robust evaluation of the learned policies. Unlike all the existing OPE estimators, *e.g.*, the DR, which only consider the estimation in the value function at current single time step, thus neglecting the average performance of a policy for a longer horizon, DW enables integration of rich previous information into the model estimation process in order to further reduce the variance. Formally, let ${\widehat{Q}}_{means}(s_t, a_t)=\frac{1}{t+1}\sum _{i=0}^{t}\gamma ^i{\widehat{Q}}(s_i, a_i)$ denote the weighted averaged value functions estimated until step *t*. The DW estimator adopts the difference between the current estimated value ${\widehat{Q}} (s_t, a_t)$ and ${\widehat{Q}}_{means}(s_t, a_t)$ to indicate how well the value functions at current step are estimated against the averaged value functions in the previous steps:

4 $$\begin{aligned} V_{DW}^{\pi _e} = \omega _{0:t} \gamma ^t \Big (r_t + {\widehat{Q}}(s_t, a_t)-{\widehat{Q}}_{means}(s_t, a_t)\Big ) \end{aligned}$$

The benefit of the DW estimator is that there is no recursive backup as in the DR estimator proposed in [22], and thus is easier to interpret and implement. We then provide the explicit form of expected value and variance of DW estimator for stochastic behavior policy $\pi _e$ and deterministic evaluation policy $\pi _b$, and analyze its upper bound bias and lower variance compared to the existing DR estimator.

**Conclusion 1**. *The expected value and variance of the DW estimator for* $\pi _e$ *can be written as*:

5 $$\begin{aligned} E(V_{DW}^{\pi _e}) = \upsilon ^{\pi _e}_{T-1} + E_{\xi }^{\pi _b}\left[ \sum _{t=1}^{T-1}\omega _{0:t}V_{t}^{back}+\sum _{t=1}^{T-1}\phi ^{t+2}\omega _{0:t}\Delta (s_t,a_t)\right] \end{aligned}$$

6 $$\begin{aligned}&nVar(V_{DW}^{\pi _e}) = E_{\xi }^{\pi _b}\sum _{t=1}^{T}{\omega _{0:t}^2}\left[ \Big (2(\Delta _2(s_t,a_t)-Q(s_t,a_t)\Big )\Big (Q(s_t,a_t) \right. \\&\left. \quad +\Delta (s_t,a_t)+r_t\Big )+\Big (\Delta _1(s_t,a_t)+r_t\Big )^2-\Big (\Delta (s_t,a_t)+r_t^2-Q(s_t,a_t)^2\Big )\right] \end{aligned}$$

where $\upsilon ^{\pi _e}_{T-1} = E_{\xi }^{\pi _b} [\sum _{t=1}^{T-1}\omega _{0:t} \gamma ^{t} r_t]$ and can be replaced by $E_{\xi }^{\pi _e} [\sum _{t=1}^{T-1} \gamma ^{t} r_t]$ under evaluation policy $\pi _e$, $V_t^{back} = r_t+\gamma r_{t-1}+\cdots +\gamma ^{t}r_0$, $\phi ^{t+2}=\gamma ^{t}-\frac{\gamma ^t+\cdots +\gamma ^0}{t}$, $\Delta (s_t, a_t) = {\widehat{Q}}(s_t, a_t)-Q(s_t, a_t)$, $\Delta _1(s_t,a_t) = Q(s_t, a_t)-Q_{means}(s_t,a_t)$, and $\Delta _2(s_t,a_t) = \Delta (s_t, a_t)-\Delta _{means}(s_t,a_t)$, where $\Delta _{means}=\frac{1}{t+1}\sum _{i=0}^{t}\gamma ^i{\Delta }(s_i, a_i)$.

#### Proof

See the Additional file 1: Appendix for a complete proof. $\square$

**Bias.** Once $E(V_{DW}^{\pi _e})$ has been computed, we can have $Bias(V_{DW}^{\pi _e}) = E(V_{DW}^{\pi _e}) - \upsilon ^{\pi _e}_{T-1} = E_{\xi }^{\pi _b}[\sum _{t=1}^{T-1}\omega _{0:t}V_{t}^{back}+\sum _{t=1}^{T-1}\phi ^{t+2}\omega _{0:t}\Delta (s_t,a_t)]$. In general, $\gamma \approx 1$, then $\phi ^{t+2} \approx 0$ and $V_t^{back} \approx r_t+r_{t-1}+\cdots +r_0$. As such, $Bias(V_{DW}^{\pi _e})$ can be approximated by $E_{\xi }^{\pi _b}[\sum _{t=1}^{T-1}\omega _{0:t}V_{t}^{back}]$, which is upper-bounded by $Bias(V_{DW}^{\pi _e}) \le T r_{t}^{max}$, where $r^{max}_t$ is the maximum positive feedback from the environment. It is clear that the upper bound bias of the DW estimator is related to the length of trajectory *T* and the maximum reward value function $r_{t}^{max}$. As the trajectory length *T* increases, the bias of the DW estimator increases linearly, indicating a complexity of *O*(*T*).

**Variance.** When $\pi _b$ is known, $\gamma = 1$ for all $s_t$ and $a_t$, $nVar(V_{DW}^{\pi _e})$ can be written as the form of Conclusion 1. For DR estimator, its variance can be given as $nVar(V_{DR}^{\pi _e})=\sum _{t=1}^{T}{\omega _{0:t}^2}[r_t^2-2Q(s_t,a_t)r_t+Q(s_t,a_t)^2+Var(Q(s_t,a_t)+\delta \Delta (s_t,a_t))]$, where $\delta = 1-\frac{\pi _b(a_t|s_t)}{\widehat{\pi _b}(a_t|s_t)} = 0$ [30]. As $\Delta (s_t,a_t)\rightarrow 0$ when the learning converges, we can get $nVar(V_{DR}^{\pi _e}) = \sum _{t=1}^{T}{\omega _{0:t}^2}[r_t^2-2Q(s_t,a_t)r_t+2Q(s_t,a_t)^2]$. From the Additional file 1: Appendix, the other form of DW variance can be written as $nVar(V_{DW}^{\pi _e}) = \sum _{t=1}^{T}{\omega _{0:t}^2}[(Q_{means}(s_t,a_t)+\Delta (s_t,a_t))^2-2*(Q_{means}(s_t, a_t)+\Delta _{means}(s_t, a_t))*\Delta (s_t,a_t)-2*(Q_{means}(s_t, a_t)+\Delta _{means}(s_t, a_t))*(Q(s_t,a_t)+r_t)]$. The difference $D{(\xi )}$ between the variances of DR and DW thus can be given as follows after some derivation:

$$\begin{aligned} D(\xi )&= \sum _{t=1}^{T}{\omega _{0:t}^2}\Big [2Q(s_t,a_t)^2+r_t^2+2\Delta _{means}(s_t, a_t)\Big (Q(s_t,a_t)\\&\quad +\Delta (s_t,a_t)+r_t\Big )-2Q(s_t,a_t)r_t+2Q_{means}(s_t, a_t)\\&\qquad \Big (Q(s_t, a_t)+r_t\Big ) -Q_{means}(s_t, a_t)^2-\Delta (s_t,a_t)^2\Big ]. \end{aligned}$$

Since $\Delta (s_t,a_t)\rightarrow 0$ and $\Delta _{means}(s_t, a_t)=\frac{1}{t+1}\sum _{i=0}^{t}\gamma ^i{\Delta }(s_i, a_i)\rightarrow 0$. $D(\xi )$ can be reduced as:

$$\begin{aligned} D(\xi )&= \sum _{t=1}^{T}{\omega _{0:t}^2}\Big [2Q(s_t, a_t)\Big (Q(s_t,a_t)-r_t\Big )+r_t^2 \\&\quad +Q_{means}(s_t, a_t)\Big (\Delta _1(s_t,a_t)+Q(s_t, a_t)+2r_t\Big )\Big ] \end{aligned}$$

It is clear that $D{(\xi )}$ depends on variables including the accumulated reward, the accumulated *Q* and $Q_{mean}$. With the convergence of RL algorithms, there are two scenarios: (1) $\sum _{t=1}^{T} r_t \ge 0$ and (2) $\sum _{t=1}^{T} r_t < 0$. In the former case, we can have $\sum _{t=1}^{T}Q(s_t,a_t) \ge 0$ and $\sum _{t=1}^{T}Q_{means}(s_t, a_t) \ge 0$. When $\gamma \approx 1$, then $Q(s_t, a_t)-r_t \approx Q(s_{t+1}, a_{t+1}) \ge 0$. Meanwhile, $\sum _{t=1}^{T} \Delta _1(s_t,a_t) = \sum _{t=1}^{T} [Q(s_t, a_t)-Q_{means}(s_t,a_t)]\>\sum _{t=1}^{T}[(Q(s_t, a_t)- \frac{1}{t}(t Q(s_t, a_t)))] = 0$. Then, we can safely get $D(\xi ) > 0$. We can also derive that $\sum _{t=1}^{T}Q(s_t,a_t) < 0$, $\sum _{t=1}^{T}Q_{means}(s_t, a_t) < 0$, and $\sum _{t=1}^{T} \Delta _{1}(s_t,a_t) <0$ hold for $\sum _{t=1}^{T} r_t < 0$ using the same calculation, and get $D(\xi ) > 0$. Based on this analysis, we can conclude that $D(\xi )$ theoretically is greater than zero, which implies that the DW estimator performs better than the DR estimator in terms of variance. We also propose the *Dueling Weight Doubly Robust* (DWDR) estimator $V_{DWDR}^ {\pi _e}$ by balancing the above two aspects. Following the DR definition in [25], which is equivalent to the recursive version in [22], we have:

7 $$\begin{aligned} V_{DWDR}^{\pi _e}&= \omega _{0:t} \gamma ^t r_t - \omega _{0:t} \gamma ^t {\widehat{Q}}(s_t, a_t) - \omega _{0:t-1} \gamma ^t {\widehat{V}}(s_t) \\&\quad + \omega _{0:t} \gamma ^t \Big (r_t + {\widehat{Q}}(s_t, a_t)-{\widehat{Q}}_{means}(s_t, a_t)\Big )\\&= \omega _{0:t} \gamma ^t \Big (2r_t - {\widehat{Q}}_{means}(s_t, a_t)\Big ) - \omega _{0:t-1} \gamma ^t {\widehat{V}}(s_t) \end{aligned}$$

### The mortality estimation process

**Fig. 2:**
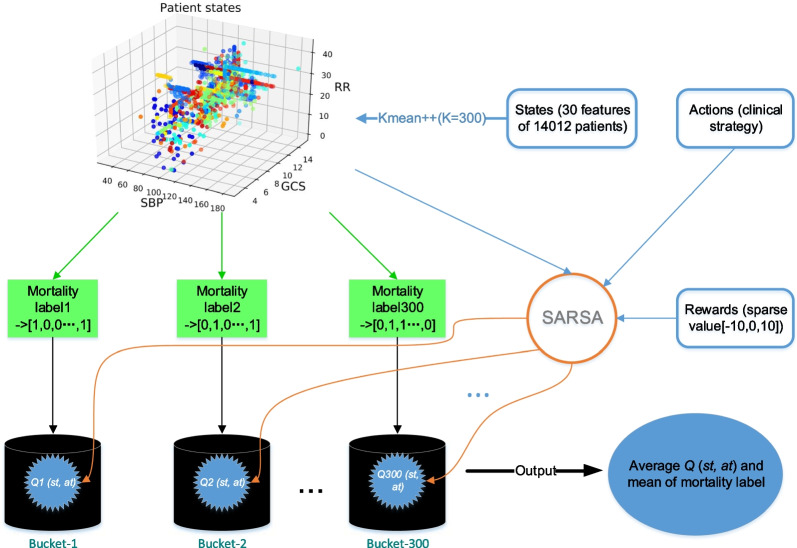
The calculation process of mortality versus *Q* values

In order to evaluate the performance (i.e., mortality) of different treatment policies, a relationship function of mortality versus expected return using the historical data should be empirically derived. Figure 2 shows the overall construction process, where $80\%$ data set is used for updating Q values using the SARSA algorithm and the remaining $20\%$ data set for estimating the mortality versus return relationship. During the update process, patient’s historical trajectories are randomly sampled to break the correlation between every tuple. To compute the Q values, the states are first clustered using k-means++ algorithm. Different values for *K* (number of clusters) were tested using the *Sum of Squared Errors* (SSE) and finally we chose $K=300$ due to a trade-off between fast descending speed and lower SSE. We further label the state of the patient as 1 if it is part of a trajectory where a patient died, and as 0 if the patient survived. The values $Q(s_t, a_t)$ are separated into discrete buckets according to different labels after state clustering. The average mortality and average $Q(s_t, a_t)$ in each bucket are then used to generate a functional relationship between the mortality and the Q values, which presents an inverse relationship, i.e., a higher expected return indicates a better policy and thus a lower mortality.

## Results

**Fig. 3:**
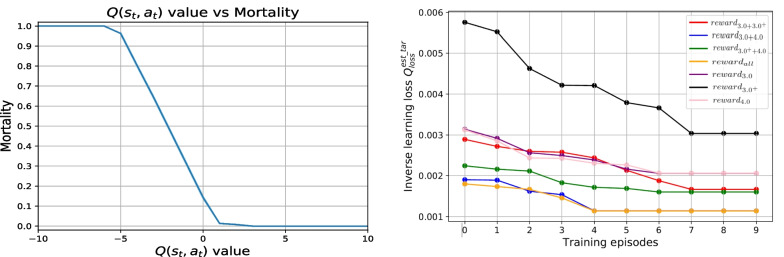
Left: The negative relationship between cumulative average $Q (s_t, a_t)$ value and mean of patients mortality; Right: The training loss using different reward functions

In Algorithm 1, we use two hidden layers of size 20, with small batch normalization for each layer. Learning rate $\alpha$ is 0.1, memory size *M* is set to 200 and batch size *B* is 32. RMSProp optimizer is applied to maximize the value functions, while SGD to optimize the weight vectors. The training process of DIRL lasts for 100 episodes, with 2000 transitions for each episode. As shown in the left subfigure in Fig. 3, as $Q (s_t, a_t)$ value increases, the average mortality of patients decreases gradually. The zero $Q (s_t, a_t)$ value of clinician strategy on the test data set corresponds to 14.6% ± 0.5% mortality, which is consistent with 14.5% mortality from the 14012 patients. The right subfigure in Fig. 3 shows the training loss of the DIRL component. It is clear that the DIRL method can infer the potentially optimal reward functions by searching the best weights among different indicators. We then compute the expected return of the final learned policy using the DR estimator and then map the result to the *mortality versus return* curve in order to get the estimated mortality, which is given by Table 4.

**Table 4:** Expected return and mortality under different polices

Policies	${\textbf{V}}_{{\textbf{DR}}}$	Mortality
$Reward_{3.0}$	$-0.0284$	14.5% ±* 0.6*%
$Reward_{3.0^+}$	$-0.1800$	17.2% ± *0.5*%
$Reward_{4.0}$	$-0.0253$	14.7% ± *0.6*%
$Reward_{3.0+3.0^+}$	0.0291	14.1% ±* 0.6*%
$Reward_{3.0+4.0}$	0.0365	13.9% ± *0.5*%
$Reward_{3.0^++4.0}$	${\textbf {0.2307}}$	**11.3%** ± *0.4*%
$Reward_{all}$	0.1546	12.2% ± *0.4*%
*Clinician*	$-0.0294$	14.5% ± *0.5*%

The bold indicates the best performance, while the italics indicate the 95% confidence interval

**Fig. 4:**
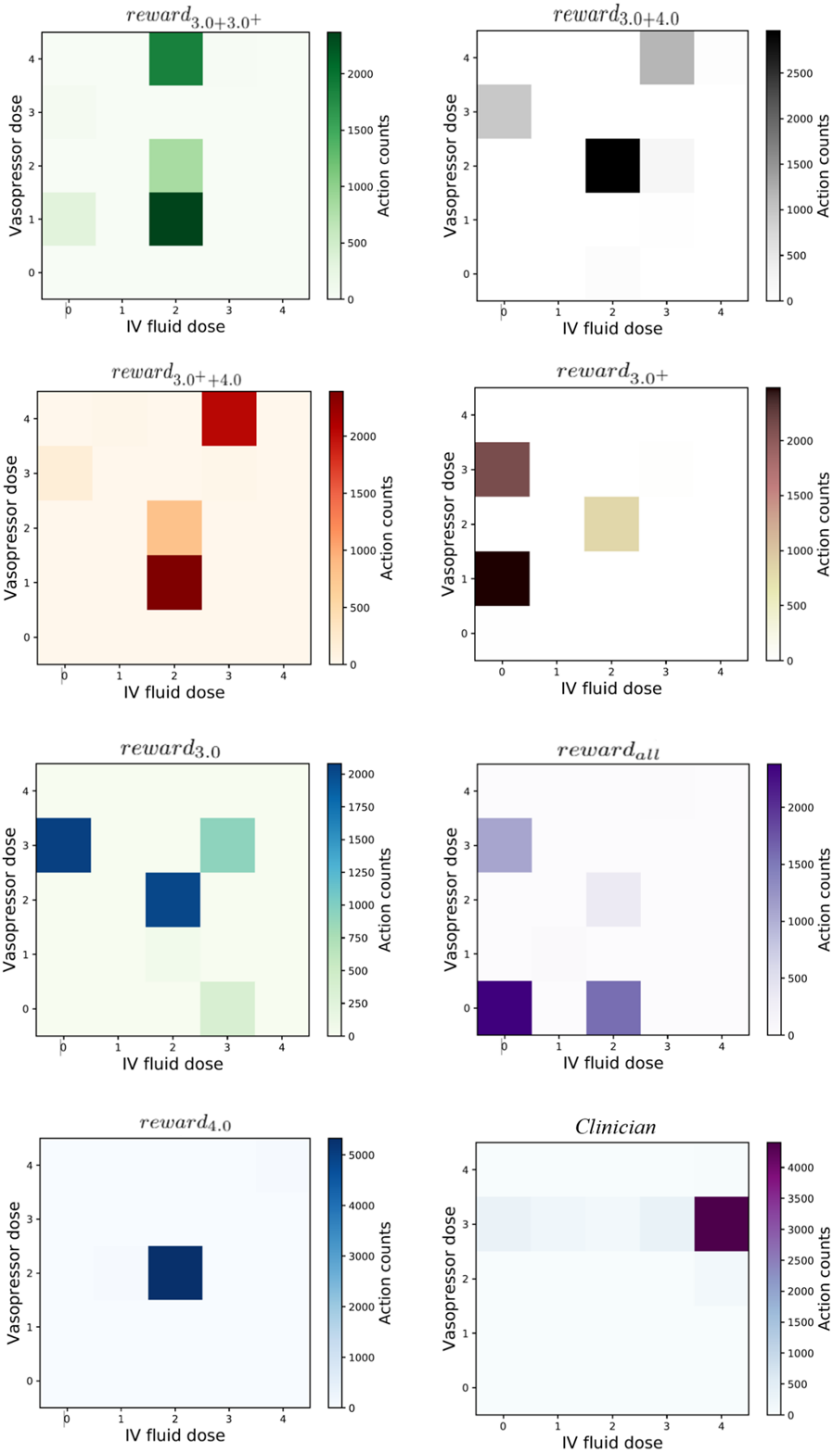
Comparison of learned strategies and the clinician strategy

**Fig. 5:**
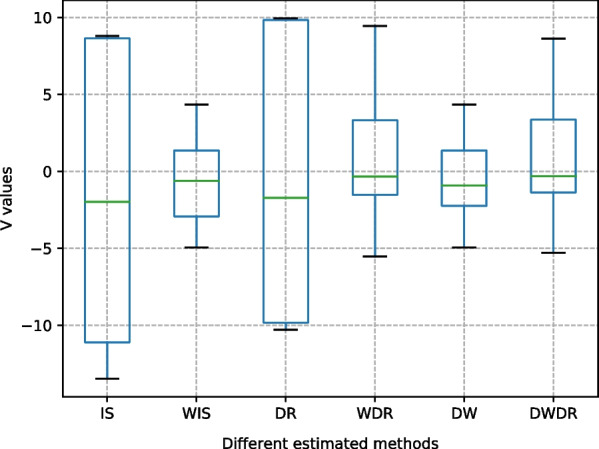
The performance of different OPE estimators

Figure 4 plots the comparison between the final learned RL strategies and the clinician strategy. Every sub-figure shows the statistical sum of every discrete action on the test data set. The dosage of a drug corresponds to the frequency the corresponding action is selected by the strategy. The result in Fig. 5 shows the effectiveness of the proposed DW estimator in evaluating the performance of the learned policies.

## Discussion

From the results, we can see that the treatment policy derived from $reward_{3.0^++4.0}$ has the highest excepted return value, with a mortality that is about $3.2\%$ lower than that of the clinician policy. This result confirms that the two indicators (PaO2 and PT) discovered by the MT component can play an important role during the treatment of sepsis patients. When these two indicators are excluded from $reward_{3.0^++4.0}$ or the $reward_{all}$ strategy, the mortality will increase by 1.9–5.9%. On the other hand, however, considering these two indicator only would also incur a mortality of $14.7\%$, which suggests that the benefits of making a balance of treatment evaluation between short-term effect and long-term mortality.

From the action distribution map in Fig. 4, we can observe that the clinician applies a higher amount of drugs in order to save the patients and action (4, 3) (corresponding to a high dosage of IV and VP) appears in the highest frequency. However, strategies of other seven reward value functions consider that the (2, 2) (corresponding to a medium dosage of IV and VP) action is more appropriate. Generally, RL recommends 40% less amount of IV fluids and 35% less amount of VP than that by the clinician, which indicates that RL will take more comprehensive consideration of the patient’s state to take drug only when it is necessary.

In terms of evaluation robustness, the results show that the IS estimator has highest variance than other estimators, which is mainly caused by the excessive cumulative importance ratio between $\pi _b$ and $\pi _e$ for a long-horizon trajectory of sepsis patient. The variance using the proposed DW estimator is superior to all alternative estimators. The significant noise introduced in the data processing process and the RL process cause a bias of IS and significant variance of DR. While DWDR has raised the variance a bit compared to DW, its bias can be further reduced, which shows the benefits of blending DW and DR to sacrifice minor variance for a better performance in bias.

## Conclusion

RL has been considered to be a promising solution to the discovery of novel treatment strategies that can potentially reduce the mortality of sepsis patients. To meet this commitment, however, more efficient and robust evaluation of the learning process as well as the final learned strategies must be properly addressed. Our work provides a critical insight that the combination of both inherent patterns in retrospective treatment data as well as the prior domain knowledge in clinical practice might be a promising way to achieve sound evaluation of treatments during learning. We also show that incorporating learning information in a longer horizon into the model estimation process helps improving the evaluation of final learned policies. Our methods have suggested some novel treatment strategies that are believed to be helpful in reducing the mortality. In our following step of work, we will conduct more comprehensive validation of our approach and seek its potential clinical applications in hospitals.

## Supplementary Information

**Additional file 1.** Complete Proof for Conclusion 1.

## Data Availability

The datasets used can be accessed freely from https://mimic.mit.edu/. The code and other materials during the current study can be available from the first author on reasonable request.
