# Supplementary material for: Towards more efficient and robust evaluation of sepsis treatment with deep reinforcement learning
Source: BMC Med Inform Decis Mak. 2023 Mar 1;23:43. doi: 10.1186/s12911-023-02126-2 (PMC9979564; doi:10.1186/s12911-023-02126-2)
Supplement: Supplementary file 1 — Additional file 1. Complete Proof for Conclusion 1. [file 12911_2023_2126_MOESM1_ESM.pdf]

Appendix

When the evaluation policy π_e is known, the DW estimator can be written as:

$$V_{DW}^{\pi_e} = \frac{1}{n}\sum_{k=1}^{n}\sum_{t=1}^{T}\gamma^t(\omega_{0:t}^{(k)}r_t^{(k)} + \omega_{0:t}^{(k)}(\widehat{Q}(s_t^{(k)}, a_t^{(k)}) - \widehat{Q}_{means}(s_t^{(k)}, a_t^{(k)}))), \quad (1)$$

where $\omega_{0:t}^{(k)} = \prod_0^t \frac{\pi_e(s_t^{(k)},a_t^{(k)})}{\pi_b(s_t^{(k)},a_t^{(k)})}$ and π_b is an uncertain policy. Then, we can compute $E_{P_\xi^{\pi_b}}[V_{DW}^{\pi_e}]$ as follows:

$$
\begin{aligned}
=&E_{P_\xi^{\pi_b}}\Big[\sum_{t=1}^{T-1}\gamma^t\big(\omega_{0:t}r_t + \omega_{0:t}\widehat{Q}^{\pi_e}(s_t,a_t) - \omega_{0:t}\frac{1}{t+1}\sum_{m=0}^{t}\widehat{Q}^{\pi_e}(s_m,a_m)\big)\Big]\\
\overset{(a)}{=}&E_{P_\xi^{\pi_b}}\Big[\sum_{t=1}^{T-1}\gamma^t\Big(\omega_{0:t}r_t + \omega_{0:t}\widehat{Q}^{\pi_e}(s_0,a_0) -\\
&\omega_{0:t}\frac{1}{t+1}\frac{(\gamma^t+\gamma^{t-1}+\cdots+\gamma^0)\widehat{Q}^{\pi_e}(s_0,a_0)-(t+1)V(s_t)}{\gamma^t}\Big)\Big]\\
=&E_{P_\xi^{\pi_b}}\Big[\sum_{t=1}^{T-1}\gamma^t\Big(\omega_{0:t}r_t + \omega_{0:t}(\frac{\widehat{Q}^{\pi_e}(s_0,a_0)-V(s_0)}{\gamma^t}) -\\
&\omega_{0:t}\big(\widehat{Q}^{\pi_e}(s_0,a_0)(\frac{\gamma^t+\gamma^{t-1}+\cdots+\gamma^0}{\gamma^t(t+1)}) - \frac{V(s_t)}{\gamma^t})\Big)\Big]\\
=&E_{P_\xi^{\pi_b}}\Big[\sum_{t=1}^{T-1}\gamma^t\omega_{0:t}r_t + \sum_{t=1}^{T-1}\Big((1-\frac{\gamma^t+\gamma^{t-1}+\cdots+\gamma^0}{t+1})\\
&\omega_{0:t}\widehat{Q}^{\pi_e}(s_0,a_0)\Big) + \sum_{t=1}^{T-1}\omega_{0:t}\big(V(s_t)-V(s_0)\big)\Big]\\
\overset{(b)}{=}&E_{P_\xi^{\pi_b}}\Big[\sum_{t=1}^{T-1}\gamma^t\omega_{0:t}r_t + \sum_{t=1}^{T-1}\Big((1-\frac{\gamma^t+\gamma^{t-1}+\cdots+\gamma^0}{t+1})\\
&\omega_{0:t}\big(Q^{\pi_e}(s_0,a_0)+\Delta^{\pi_e}(s_0,a_0)\big)\Big) + \sum_{t=1}^{T-1}\omega_{0:t}V_t^{back}\Big]\\
\overset{(c)}{=}&\upsilon_{T-1}^{\pi_e} + E_{P_\xi^{\pi_b}}\Big[\sum_{t=1}^{T-1}\omega_{0:t}\underbrace{\phi^t}_{\approx 0}\big(Q^{\pi_e}(s_0,a_0)+\Delta^{\pi_e}(s_0,a_0)\big)+\\
&\sum_{t=1}^{T-1}\omega_{0:t}V_t^{back}\Big]\\
=&\upsilon_{T-1}^{\pi_e} + E_{P_\xi^{\pi_b}}\Big[\sum_{t=1}^{T-1}\omega_{0:t}V_t^{back}\Big],
\end{aligned}
$$

where $V_t^{back} = V(s_t) - V(s_0)$, $\phi^t = 1 - \frac{\gamma^t+\gamma^{t-1}+\cdots+\gamma^0}{t+1}$ and $\upsilon_{T-1}^{\pi_e} = E_\xi^{\pi_b}[\sum_{t=1}^{T-1}\omega_{0:t}\gamma^t r_t]$. Therefore, the bias of the DW estimator can be written as: $Bias(V_{DW}^{\pi_e}) = E_{P_\xi^{\pi_b}}(V_{DW}^{\pi_e}) - \upsilon_{T-1}^{\pi_e} = E_{P_\xi^{\pi_b}}[\sum_{t=1}^{T-1}\omega_{0:t}V_t^{back}]$.

(a) This derivation is based on $\widehat{Q}^{\pi_e}(s_t,a_t) = \gamma\widehat{Q}^{\pi_e}(s_{t+1},a_{t+1}) + r_t$, which is transformed into $\widehat{Q}^{\pi_e}(s_{t+1},a_{t+1}) = \frac{\widehat{Q}^{\pi_e}(s_t,a_t)-r_t}{\gamma}$, and then $\widehat{Q}^{\pi_e}(s_t,a_t) = \frac{\widehat{Q}(s_0,a_0)-(r_t+\gamma r_{t-1}+\cdots+\gamma^t r_0)}{\gamma^t}$.

(b) We denote $\Delta^{\pi_e}(s_t, a_t) = \widehat{Q}^{\pi_e}(s_t, a_t) - Q^{\pi_e}(s_t, a_t)$.
(c) This derivation is based $(i) \upsilon^{\pi_e}_{T-1} = E_{s_1 \sim P_1}[V^{\pi_e}(s_1)]$, and
$(ii) \phi^t = 1 - \frac{\gamma^t + \gamma^{t-1} + \cdots + \gamma^0}{t+1} \approx 0$ under the condition of $\gamma \approx 1$. Next, we give a proof of the variance $nVarP^{\pi_b}_{\xi}(\rho^{\pi_e}_{DW})$ of the DW estimator. For convenience, we omit the notation time t on the variance. Then, $nVarP^{\pi_b}_{\xi}(\rho^{\pi_e}_{DW})$ can be computed as follows:

$$
\begin{aligned}
&= E_{P^{\pi_b}_{\xi}}\Big[\frac{\pi_e(a|s)}{\pi_b(a|s)}\Big(Q(s,a) + \Delta(s,a) - Q_{means}(s,a) - \\
&\quad \Delta_{means}(s,a) + r\Big)^2\Big] - E_{P^{\pi_b}_{\xi}}\Big[\frac{\pi_e(a|s)}{\pi_b(a|s)}(\Delta(s,a) + r + Q(s,a))\Big]^2 \\
&= E_{P^{\pi_b}_{\xi}}\Big[\Big(\frac{\pi_e(a|s)}{\pi_b(a|s)}(Q(s,a) + r)\Big)^2\Big] + E_{P^{\pi_b}_{\xi}}\Big[\Big(\frac{\pi_e(a|s)}{\pi_b(a|s)}(\Delta(s,a)\Big)^2\Big] \\
&\quad + E_{P^{\pi_b}_{\xi}}\Big[\Big(\frac{\pi_e(a|s)}{\pi_b(a|s)}(Q_{means}(s,a) + \Delta(s,a))\Big)^2\Big] + \\
&\quad 2E_{P^{\pi_b}_{\xi}}\Big[\Big(\frac{\pi_e(a|s)}{\pi_b(a|s)}\Big)^2(Q(s,a) + r)\Delta(s,a)\Big] \\
&\quad - 2E_{P^{\pi_b}_{\xi}}\Big[\Big(\frac{\pi_e(a|s)}{\pi_b(a|s)}\Big)^2(Q_{means}(s,a) + \Delta_{means}(s,a))\Delta(s,a)\Big] \\
&\quad - 2E_{P^{\pi_b}_{\xi}}\Big[\Big(\frac{\pi_e(a|s)}{\pi_b(a|s)}\Big)^2(Q_{means}(s,a) + \Delta_{means}(s,a))\big(Q(s,a) + r\big)\Big] \\
&\quad - E_{P^{\pi_b}_{\xi}}\Big[\Big(\Delta(s,a)\frac{\pi_e(a|s)}{\pi_b(a|s)}\Big)^2\Big] - E_{P^{\pi_b}_{\xi}}\Big[\big(Q(s,a) + r\big)\Big(\frac{\pi_e(a|s)}{\pi_b(a|s)}\Big)^2\Big] \\
&\quad - 2E_{P^{\pi_b}_{\xi}}\Big[\big(r + Q(s,a)\big)\Delta(s,a)\Big(\frac{\pi_e(a|s)}{\pi_b(a|s)}\Big)^2\Big]. \\
&= E_{P^{\pi_b}_{\xi}}\Big[\Big(\frac{\pi_e(a|s)}{\pi_b(a|s)}\Big)^2\Big(Q_{means}(s,a) + \Delta(s,a)\Big)^2\Big] - 2E_{P^{\pi_b}_{\xi}}\Big[\Big(\frac{\pi_e(a|s)}{\pi_b(a|s)}\Big)^2 \\
&\quad \Big(Q_{means}(s,a) + \Delta_{means}(s,a)\Big)\Delta(s,a)\Big] - 2E_{P^{\pi_b}_{\xi}}\Big[\Big(\frac{\pi_e(a|s)}{\pi_b(a|s)}\Big)^2 \\
&\quad \Big(Q_{means}(s,a) + \Delta_{means}(s,a)\Big) * \Big(Q(s,a) + r)\Big)\Big] \\
&\overset{(a)}{=} E_{P^{\pi_b}_{\xi}}\Big[\Big(\frac{\pi_e(a|s)}{\pi_b(a|s)}\Big)^2\Big(\Delta_1(s,a)^2 + 2\Delta_2(s,a)\big(Q(s,a) + \Delta(s,a) + r\big) \\
&\quad + 2\Delta_1(s,a)r - Q(s,a)^2 - 2Q(s,a)\Delta(s,a) - 2Q(s,a)r \\
&\quad - 2\Delta(s,a)r - \Delta(s,a)^2\Big)\Big] \\
&\overset{(b)}{=} E_{P^{\pi_b}_{\xi}}\Big[\Big(\frac{\pi_e(a|s)}{\pi_b(a|s)}\Big)^2\Big(\Delta_1(s,a)^2 + 2\Delta_2(s,a)\big(Q(s,a) + r\big) \\
&\quad + 2\Delta_1(s,a)r - Q(s,a)^2 - 2Q(s,a)r\Big)\Big] \\
&= E^{\pi_b}_{\xi}\Big[\omega^2_{0:t}\Big(2\big(\Delta_2(s,a) - Q(s,a)\big)\big(Q(s,a) + r\big) \\
&\quad + \big(\Delta_1(s,a) + r\big)^2 - r^2 - Q(s,a)^2\Big)\Big]
\end{aligned}
$$

where $\omega_{0:t} = \prod_0^t \frac{\pi_e(s,a)}{\pi_b(s,a)}$. By adding the notation time t, this formula is equivalent to the result in Conclusion 1 in the main file.
(a) We denote $\Delta_1(s,a) = Q(s,a) - Q_{means}(s,a)$ and
$\Delta_2(s,a) = \Delta(s,a) - \Delta_{means}(s,a)$.

(b) When the algorithm converges, we can conclude that $\widehat{Q}(s_t, a_t)$ is an unbiased estimate of $Q(s_t, a_t)$, and thus $\Delta(s_t, a_t) = \widehat{Q}(s_t, a_t) - Q(s_t, a_t) \approx 0$.
